# Comparing Distal-Jet with Dental Anchorage to Distal-Jet with Skeletal Anchorage: A Prospective Parallel Cohort Study

**DOI:** 10.3390/dj10100179

**Published:** 2022-09-27

**Authors:** Federica Altieri, Martina Mezio, Rosanna Guarnieri, Michele Cassetta

**Affiliations:** Department of Oral and Maxillofacial Sciences, Sapienza University of Rome, 00161 Rome, Italy

**Keywords:** distal-jet appliance, skeletal anchorage, computer-assisted, miniscrew, orthodontics

## Abstract

The use of traditional intra-oral devices in maxillary molar distalization is not without undesirable consequences. The aim of the present study was to compare the miniscrew-supported distal-jet appliance to a traditional distal-jet appliance by evaluating the amount of upper first molar distalization and the dentoalveolar side effects. Data of 600 subjects visited at the orthodontic unit of Sapienza University of Rom were analyzed. Only 46 patients met the inclusion criteria and were selected and treated. Subjects were assigned randomly to receive treatment either with miniscrew-supported distal-jet appliance (Group A) or with a traditional distal-jet appliance (Group B). In Group A, miniscrews were inserted using a computer-guided surgical guide. The amount of upper first molar distalization and the dentoalveolar side effects were assessed both on the digital casts and on the lateral cephalometric radiograph at the end of the distalization phase. A descriptive statistical analysis that included the mean values and the standard deviation was conducted to evaluate the molar distalization and the dentoalveolar effects in two groups. Intergroup differences were determined using the Student’s *t*-test. The significance was set at *p* ≤ 0.05. In Group A, greater maxillary first molar distalization and a spontaneous distalization of the first premolars and a palatal inclination of central incisors were observed. By contrast, in Group B, the first premolars tipped mesially and a proclination of the maxillary central incisors was observed. In both groups, the transverse widths of the dental arch increased while a greater tendency of first premolar extrusion and of maxillary first molar rotation was observed in Group B. The skeletal anchorage device achieved greater first molar distalization and did not cause dento-alveolar side effects.

## 1. Introduction

Correction of Class II malocclusion by distalization of maxillary molars is a non-extraction treatment approach, commonly used in orthodontic clinical practice for the upper arch space recovery. Traditional treatment of Class II, with intra- and/or extra-oral devices, often requires an active patient collaboration [1,2,3,4]. Extra-oral traction, although it is an effective treatment, is aesthetically unacceptable and requires patient compliance [1]. Intra-oral devices (for example Pendulum and distal-jet) [2,3,4] even though they do not require the patient’s cooperation, produce side effects such as the mesial migration of the premolars and incisors [2,3,4]. 

To prevent loss of anchorage, it is now possible to use skeletal support devices, i.e., orthodontic devices that are temporarily and directly fixed to the alveolar bone by means of orthodontic miniscrews, which are extensively used by orthodontists for tooth movement in conventional orthodontic procedures such as molar protraction, canine retraction, correction of dental midline, space closure, and distalization of maxillary molars [5,6,7]. The miniscrew-supported distal-jet appliance is an example [5,6].

Nowadays, thanks to three-dimensional (3D) printing with biocompatible resins, several orthodontic procedures can be simplified [7,8]. The 3D printed surgical guide allows the easy and safe orthodontic miniscrew insertion.

The purpose of this prospective parallel cohort study was to study the dento-alveolar effects of two different types of devices for molar distalization:

− a traditional distal-jet appliance;
− a miniscrew-supported distal-jet appliance.

It was hypothesized that the miniscrew-supported distal-jet appliance causes a greater amount of molar distalization and does not cause dentoalveolar side effects such as mesial drift of the premolar teeth and upper incisor flaring.

## 2. Materials and Methods

This prospective parallel cohort study was conducted at the Department of Oral and Maxillofacial Sciences, School of Dentistry, Sapienza, University of Rome, Orthodontics Unit, between September 2018 and December 2020.

The study was performed in accordance with the ethical principles of the Declaration of Helsinki. Parents or guardians were informed of the content, risks, and benefits of the study and written consent was obtained. 

In total, 600 subjects were examined in six months (March 2019–May 2019), only 46 met the following inclusion criteria:

- Adolescents with late mixed dentition or permanent dentition;
- Good oral hygiene with the absence of plaque or calculus;
- Bilateral dental Class II malocclusion with mesial migration of the maxillary posterior teeth;
- No adequate space for the correct eruption of one or both maxillary canines;
- Absence of previous orthodontic treatment;
- No systemic syndrome involved.

**Figure 1:**
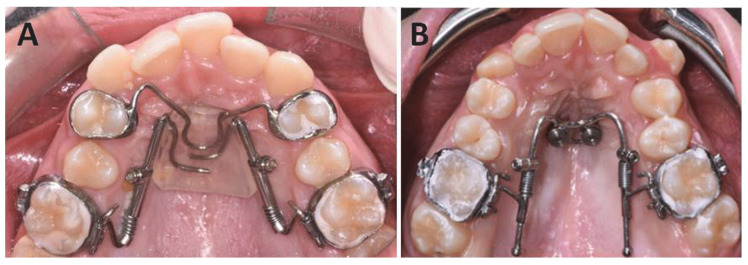
(**A**) Dental-anchored distal-jet; (**B**) distal-jet with skeletal anchorage.

Subjects were assigned randomly to receive treatment with a miniscrew-supported distal-jet, in Group A, or with a traditional distal-jet in Group B (Figure 1A,B). 

**Figure 2:**
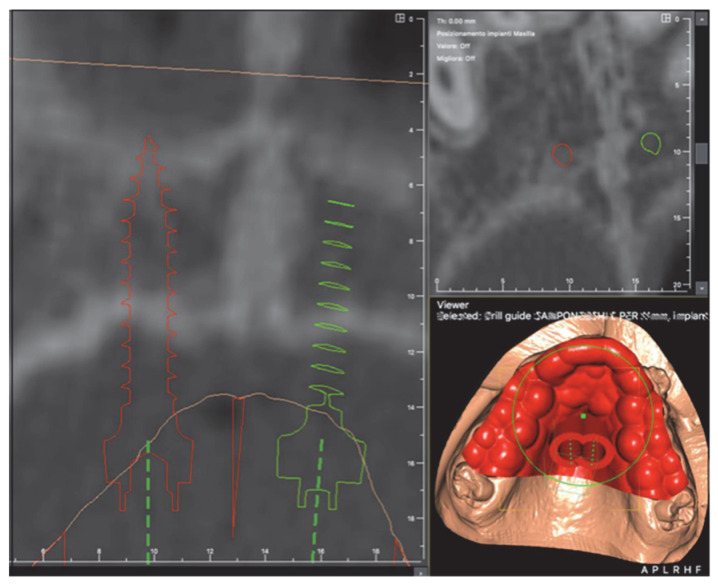
Insertion phase of the orthodontic miniscrews by surgical template.

A randomization sequence was created using Clinstat statistical software (Martin Bland, York, UK) for the treatment allocation of the two groups. The insertion of the orthodontic miniscrews was computer-guided, thanks to the use of a surgical guide made with a 3D printer (Stratasys OrhoDesktop; Stratasys, Rehovot, Israel). The insertion sites of the miniscrews were planned on the 3D images, created by matching the STL files of the maxillary bone bases–obtained from the CBCT exam–and digital model files of the dental arches (Figure 2). 

**Figure 3:**
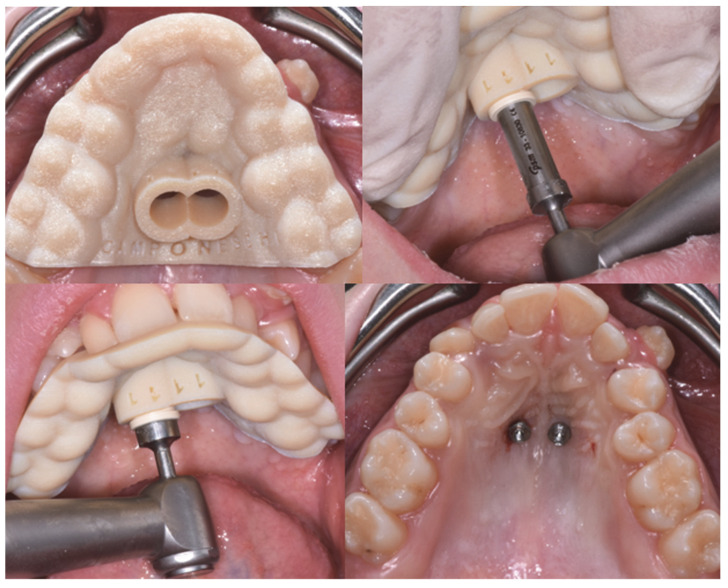
Insertion phase of the orthodontic mini-screws by surgical template.

The length and diameter of the miniscrews used (BENEfit, PSM medical solutions, Tuttlingen, Germany) were determined in the planning phase (lengths: 7, 9, 11, 13 mm; diameters: 2.0, 2.3 mm). In the current study, the orthodontic device was applied simultaneously with the insertion of the miniscrews in a single outpatient session (Figure 3).

**Figure 4:**
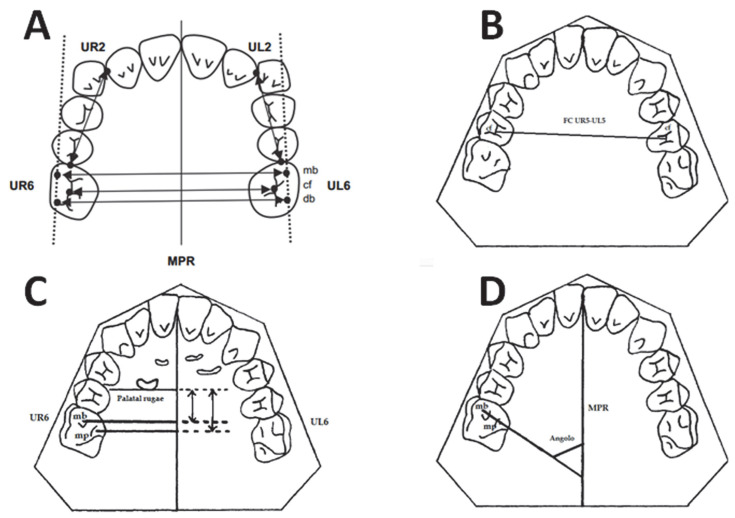
(**A**) Distance between upper lateral incisor (left: UL2; right: UR2) and first maxillary molar (left: UL6; right: UR6); inter-molar distance from the mesio-buccal cusp (MB) tip of the UR6 to the mesio-buccal cusp tip of the UL6; the distance from the disto-buccal (DB) cusp tip of the UR6 to the disto-buccal cusp tip of the UL6; the distance from the lowest point of central fossa (CF) of the UR6 to the lowest point of central fossa of the UL6; (**B**) premolar transversal variation; (**C**) molar distalization- third palatal rugae: line AR,BR (right) line AL,BL (left); (**D**) angle of molar rotation.

The distal-jet devices were activated in both groups by the compression of a super-elastic Ni-Ti helical spring that releases a force of about 250 N, the force necessary to obtain the molar displacement. The coil spring was reactivated every 4 weeks until bilateral molar class I was reached. The digital casts of the dental arches before treatment (T0) and at the end of treatment (T1) were created and analyzed. The lateral cephalometric radiograph (LCR) was requested pre-treatment (T0) and at the end of therapy (T1). On LCR, cephalometric analysis was performed using the Oris Ceph software (Oris Ceph, Elite Computer, Vimodrone, Milan, Italy). The amount of upper molar distalization and the dentoalveolar side effects were evaluated both on the dental casts and on LCR. On the digital casts, the measurements were carried out following the method of Haas and Cisneros, Hoggan and Sadowsky, and Kinzinger et al. [9,10,11,12] (Figure 4).

**Figure 5:**
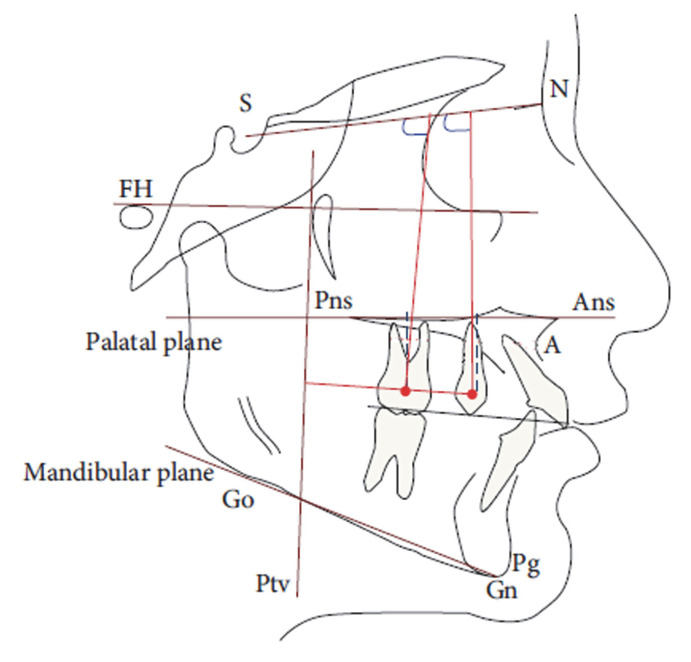
Cephalometric measurements with the reference landmarks.

The measurements performed on the LCR are shown in Figure 5 and Table 1.

A database was created using Excel (Microsoft, Redmond, WA, USA), with appropriate checks to identify errors. Data were analyzed using Python 3.6 Statistical analysis software. A descriptive statistical analysis was conducted on the differences between pre- and post- therapy values, i.e., mean values and standard deviation, to evaluate the amount of distalization of the upper molar and the dento-alveolar side effects, recorded at the end of the phase of distalization. The Student’s t-test was used to analyze the differences between the two groups. Statistical significance was set with a *p* ≤ 0.05. All measurements were performed twice, within a monthly interval, by the same calibrated examiner (F.A). To calculate the intra-operator reliability, all measurements were repeated by the same examiner (F.A) after 4 weeks. The intra-operator reliability was evaluated using Choen’s Kappa, a statistical coefficient that indicates the degree of accuracy and reliability in a statistical classification; a value close to 1 indicates agreement. Good intra-operator reliability was found with an average value of Choen’s Kappa coefficient equal to K = 0.91. On the basis of the results of a previous study, it was considered a mean molar distalization of 5.3 ± 2.1 mm for group A and a mean of 0.9 ± 0.9 mm for group B [13]. Considering a power of 95% and a Type I error of 0.05. These considerations determine an acceptable sample size value for a future study of at least 8 patients (4 for each group).

## 3. Results

Forty-six subjects (20 males and 26 females) were included in the study and treated. The subjects were divided into two groups: twenty-two subjects were assigned to receive distal-jet treatment with miniscrews (Group A) and 24 were treated with a traditional distal-jet (Group B). The two groups were comparable considering the age (mean 13.2 years 1.7 SD) and the characteristics of the malocclusion. No miniscrew was lost during the treatment.

The mean values and the standard deviation (SD) of the difference between the pre and post treatment measurements (T1–T0) are shown in Table 2.

The distance from the distal point of contact of the lateral incisor to the mesial point of contact of the first molar (Figure 4A) increased in both groups. In Group A, the first upper molars distalized by an average of 4.3 mm for the AR line and 4.1 mm for the AL line, while a lesser distalization occurred in Group B with an average of 1.5 mm for the AR line and 3.1 mm for the AL line (Table 2). In both groups, a comparable increase in the measured transverse diameter was observed (Table 2), indicating an expansion of the dentoalveolar arch. As for the molar rotation, in Group B, there was greater rotation of the first permanent molars when compared to Group A (Table 2). The measurements performed at T0 and T1 on LCRs are shown in Table 3. These measurements showed a greater distalization of the first upper molar in Group A (PTV-U6 = −3.9 mm) compared to Group B (PTV-U6 = −2.2 mm). 

On average there was a distal tipping of the first upper molar (SN-U6) of −0.1° + 3 in Group A and −2.5° + 6.8 in Group B. In Group A, the first premolars underwent a distalization of 2.2 mm + 1.5 with an inclination of −2.4° + 2.4 (SN ^ U4). By contrast, in Group B, the first premolars mesialized by 2.2 mm + 1.9 and a mesial inclination of 3.6° + 4.7 was found. On the vertical plane, no changes were found at the molar level. In Group A, the first premolars did not undergo any alteration in the vertical plane (PP-U4), while in Group B there was an average extrusion of 1.1 mm + 0.8 (Table 2).

In Group B, the upper central incisors were buccal-inclined on average by 5.5° + 4.0 with respect to the palatal plane (PP ^ U1) and by 5.3° + 4.6 with respect to the anterior cranial base (SN ^ U1), while in Group A, palatal inclination of the central incisors was observed (PP ^ U1 = −3.4° + 2.8; SN ^ U1 = −4.9° + 3.9) with a statistically significant difference for both values (*p* = 0.00). An increase in the interincisal angle (1 ^ 1) was found in Group A and a reduction in Group B (Table 2). At the end of the treatment, the overjet was slightly reduced in Group A but it increased in Group B. A greater reduction in overbite was found in Group B (OVB = −0.9 mm + 1) as compared to Group A (OVB = −0.2 mm + 0.9). The vertical (FMA, PP-GO-ME, SN-GO-ME) and sagittal (ANB) dimensions remained almost unchanged in both groups (Table 3).

**Figure 6:**
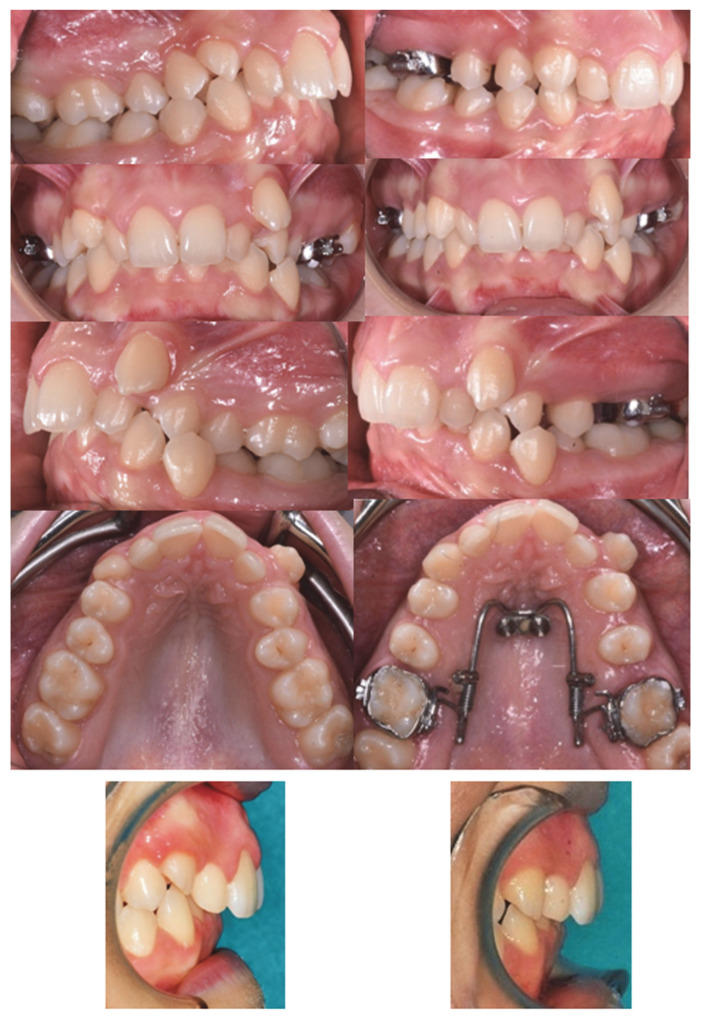
Intraoral images: distal-jet with skeletal anchorage pre-treatment (T0) (**left**) and post-treatment (T1) (**right**).

**Figure 7:**
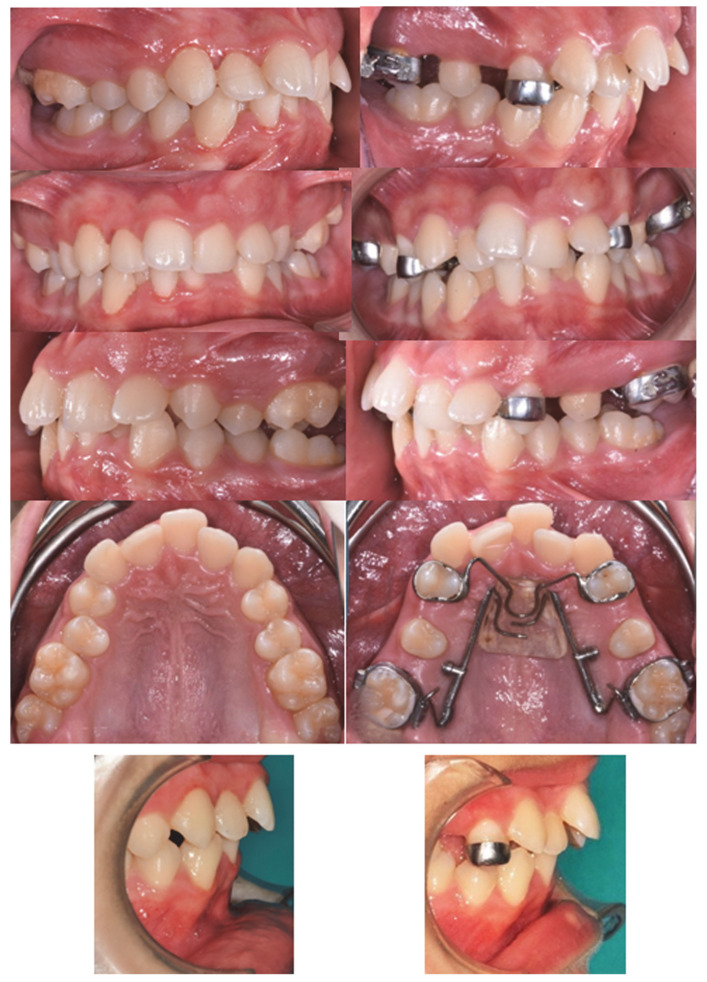
Intraoral images; distal-jet with dental anchorage pre-treatment (T0) (**left**) and post-treatment (T1) (**right**).

Figure 6 and Figure 7 show intra-oral, pre-treatment (T0), and post-treatment (T1) images of the miniscrew-anchored distal-jet and the dental-anchored distal-jet.

## 4. Discussion

This prospective parallel cohort study, conducted on 46 subjects, compared two maxillary molars distalization systems: the skeletally anchored distal-jet and the dental-anchored distal-jet. The present results confirmed the initial hypothesis: the miniscrew-supported distal-jet determines a greater molar distalization and the absence of premolars mesial drift and incisors flaring. 

In both groups, an increase of the distance from the distal point of contact of the lateral incisor to the mesial point of contact of the first molar (UR2-UR6, UL2-UL6, called support zone), measured on the digital cast, was observed. This result agrees with the study results of Kinzinger et al. [11,12]; however, this measurement should be interpreted with caution; the position of the lateral incisors can be modified, during the orthodontic treatment in subjects with mixed dentition, by the physiological eruption of the permanent canines. Instead, the position of the third palatine rugae (AR, BR, AL, BL), used as an anterior landmark, is not affected by the dentition process or by orthodontic treatment and it can be considered a stable and reliable point [13]. These measurements (AR, BR, AL, BL), showed a greater molar distalization in Group A compared to Group B. When the molar distalization was evaluated on LCR, through the PTV-U6 distance, the increase of the support zone in the group with the skeletal distal-jet was determined by molar distalization, whereas a combination of molar distalization and loss of anterior anchorage was observed in subjects treated with a dentally-anchored distal-jet. According to Bolla et al. [14] and Ngantung et al. [15] the traditional distal-jet is an efficient orthodontic device for the class II malocclusion correction; however, the force generated by the compression of the NiTi spring produces about 71% of molar distalization and 29% of loss of anterior anchorage with a mesialization of the first premolar [14,15]. In the present study, an increase of PTV-U4 distance was observed in subjects treated with the traditional distal-jet and a reduction in subjects treated with skeletal anchorage devices (PTV-U4 = −2.2 mm). In agreement with the literature, the distal premolars movement, observed in Group A, could be explain by the absence of bands on these teeth with a spontaneous distal migration due to the transeptal fibers activation. In contrast, in Group B, a mesial migration of the banded first premolars was observed [16].

Regarding the first molar inclination (SN^U6), a lower inclination was observed in subjects with a skeletal distal-jet, but this result could be influenced by the presence or absence of upper second molars rather than the type of orthodontic device. As stated by Kinzinger et al. [12] the degree of distal tipping of first molars is less in patients with erupted second molars than in those whose second molars are not yet erupted.

Concerning the presence of maxillary molar rotation, this phenomenon was observed also by other authors [11,12,17], with a biomechanical explanation: when the force is applied palatally to the center of resistance of the molars, a rotation of these teeth occurs.

The premolars extrusion was observed in subjects treated with the skeletal distal-jet since these teeth were not included in the anchorage unit [11,12,17].

An increase in the proclination of the upper incisors was found in subjects treated with the traditional distal-jet, whereas a palatal inclination occurred in subjects treated with the skeletally anchored distal-jet (PP ^ U1; SN ^ U1; 1 ^ 1); these results are in agreement with the literature [6,17,18]. Only one study described a slight proclination of the upper central incisors after the use of the miniscrew-supported distal-jet [12], but the different design of the orthodontic device used (that is, the presence/absence of occlusal rests on the first premolars), could explain this difference.

## 5. Conclusions

In conclusion, both devices can be considered effective in the correction of the Class II malocclusion. The miniscrew-supported distal-jet allows for greater distalization at the molar level without causing loss of anterior anchorage. Spontaneous distalization of the first premolars and a palatal inclination of the incisors were also observed. The traditional distal-jet determines a lower amount of distalization on the molars, which is associated with a loss of anterior anchorage with mesialization and extrusion of the first premolars and a labial-inclination of the upper incisors.

## Figures and Tables

**Table 1:** Cephalometric measurements.

Cephalometric Variables	Description
PTV-U6 (mm)	Horizontal measurement from the PTV line to the maxillary first molar.
PTV-U4 (mm)	Horizontal measurement from the palatal plane to the maxillary first premolar.
PP-U6 (mm)	Vertical measurement from the palatal plane to the maxillary first molar.
PP-U4 (mm)	Vertical measurement from the palatal plane to the maxillary first premolar.
SN ^ U6 (degrees °)	Angle formed by the intersection of the axis of the first upper molar and the Sella-Nasion plane.
SN ^ U4 (degrees °)	Angle formed by the intersection of the axis of the first upper premolar and the Sella-Nasion plane
PP ^ U1 (degrees °)	Angle between the upper central incisor and the palatal plane
SN ^ U1 (degrees °)	Angle between the upper central incisor and the anterior cranial base
1 ^ 1 (degrees °)	Interincisive angle
ANB (degrees °)	Angle used for the evaluation of the skeletal class in the sagittal plane
FMA (degrees °)	Angle between the Frankfurt plane and the Gonion-Menton plane
PP ^ Go-Me (degrees °)	Angle between the palatal plane and the mandibular plane
SN ^ Go-Me (degrees °)	Angle between the anterior cranial base and the mandibular plane

**Table 2:** Linear and angular measurements carried out on the study model. For T1−T0 (value after distalization)—(value before distalization); SD: Standard Deviation; NS: Not Significant; * *p* value ≤ 0.05; CI: confidence interval.

Variable	T1−T0 Group A	T1−T0 Group A SD	T1−T0 Group B	T1−T0 Group B SD	Significance	Mean Difference (95% CI)
UR2-UR6	4.2	1.7	3.7	2.7	NS	0.50 (−1.44 to 2.44)
UL2-UL6	2.9	2.2	4	2.3	NS	−1.08 (−3.17 to 1.01)
MB UR6-UL6	1.9	2.4	3.2	1.3	NS	−1.25 (−3.16 to 0.66)
CF UR6-UL6	1.9	1.9	3.7	2.0	*	−1.77 (−3.53 to −0.02)
DB UR6-UL6	3	3.4	3.8	1.4	NS	−0.76 (−3.42 to 1.90)
UR6 ROTATION	1.5	6.7	−2.7	5.1	NS	4.25 (−1.50 to 10.00)
UL6 ROTATION	−0.3	7.4	−1.6	6.0	NS	1.23 (−5.18 to 7.65)
CF UR5-UL5	1.4	1.9	2.7	2.9	NS	−1.17 (−3.01 to 0.67)
LINE AR	4.3	2.8	1.5	3.1	*	2.85 (0.17 to 5.52)
LINE AL	4.1	2.8	3.1	3.3	NS	0.95 (−1.81 to 3.71)
LINE BR	3.8	2.8	1.0	2.4	*	2.87 (0.41 to 5.3)
LINE BL	3.6	2.5	2.5	2.7	NS	1.03 (−1.37 to 3.42)

**Table 3:** Cephalometric analysis results for T1−T0 (value after distalization)—(value before distalization); SD: Standard Deviation; NS: Not Significant; * *p* value ≤ 0.05; CI: confidence interval.

Variable	T1−T0 Group A	T1−T0 Group A SD	T1−T0 Group B	T1−T0 Group B SD	Significance	Mean Difference (95% CI)
PTV-U6 mm	−3.9	1.2	−2.2	1.5	*	−1.72 (−2.91 to −0.53)
PTV-U4 mm	−2.2	1.5	2.2	1.9	*	−4.45 (−5.98 to −2.92)
SN ^ U6	−0.1	3.0	−2.5	6.8	NS	2.38 (−1.96 to 6.73)
SN ^ U4	−2.4	2.4	3.6	4.7	*	−5.98 (−9.10 to −2.87)
PP-U6 mm	0.6	3.6	0.3	1.0	NS	0.22 (−2.52 to 2.96)
PP-U4 mm	−0.1	0.6	1.1	0.8	*	−1.19 (−1.82 to −0.56)
PP ^ U1	−3.4	2.8	5.5	4.0	*	−8.82 (−11.82 to −5.82)
SN ^ U1	−4.9	3.9	5.3	4.6	*	−10.25 (−14.10 to −6.40)
1 ^ 1	3.9	4.5	−5.1	6.9	*	8.95 (3.90 to 14.00)
OVJ	−0.6	0.5	1.0	0.6	*	−1.55 (−2.02 to −1.07)
OVB	−0.2	0.9	−0.9	1	NS	0.75 (−0.10 to 1.61)
ANB	−0.3	0.8	0.1	0.7	NS	−0.16 (−0.85 to 0.52)
FMA	−0.6	2.5	0.0	3.2	NS	−0.65 (−3.21 to 1.91)
PP ^ Go-Me	−0.9	2.2	0.3	2.6	NS	−1.26 (−3.42 to 0.89)
SN ^ Go-Me	−0.4	2.1	0.1	1.8	NS	−0.50 (−2.37 to 1.38)

## Data Availability

Not applicable.
